# Platelet activation in critically ill COVID-19 patients

**DOI:** 10.1186/s13613-021-00899-1

**Published:** 2021-07-17

**Authors:** Nader Yatim, Jeremy Boussier, Richard Chocron, Jérôme Hadjadj, Aurélien Philippe, Nicolas Gendron, Laura Barnabei, Bruno Charbit, Tali-Anne Szwebel, Nicolas Carlier, Frédéric Pène, Célia Azoulay, Lina Khider, Tristan Mirault, Jean-Luc Diehl, Coralie L. Guerin, Frédéric Rieux-Laucat, Darragh Duffy, Solen Kernéis, David M. Smadja, Benjamin Terrier

**Affiliations:** 1 Translational Immunology Lab, Department of Immunology, Institut Pasteur, 75015 Paris, France; 2 Department of Internal Medicine, National Reference Center for Rare Systemic Autoimmune Diseases, AP-HP, APHP.CUP, Hôpital Cochin, 75014 Paris, France; 3 Imagine Institute Laboratory of Immunogenetics of Pediatric Autoimmune Diseases, INSERM, UMR 1163, Université de Paris, 75015 Paris, France; 4 Innovative Therapies in Haemostasis, INSERM, Université de Paris, 75006 Paris, France; 5 Hematology Department, APHP-CUP, 75015 Paris, France; 6 Biosurgical Research Lab (Carpentier Foundation), Georges Pompidou European Hospital, 75015 Paris, France; 7 Cytometry and Biomarkers UTechS, CRT, Institut Pasteur, 75015 Paris, France; 8 Department of Pulmonology, APHP-CUP, Hôpital Cochin, 75014 Paris, France; 9 Université de Paris, Institut Cochin, INSERM U1016, CNRS UMR8104, 75006 Paris, France; 10 Service de Médecine Intensive and Réanimation, APHP-CUP, Hôpital Cochin, 75014 Paris, France; 11 Vascular Medicine Department, APHP-CUP, Université de Paris, 75015 Paris, France; 12 Université de Paris, INSERM, U970, PARCC, Paris, France; 13 Intensive Care Unit, APHP-CUP, 75015 Paris, France; 14 Emergency Department, APHP-CUP, 75015 Paris, France; 15 Equipe Mobile d’Infectiologie, APHP-CUP, Hôpital Cochin, 75014 Paris, France; 16 Epidemiology and Modelling of Antibiotic Evasion (EMAE), Institut Pasteur, 75015 Paris, France; 17 Université de Paris, INSERM, IAME, Université de Paris, 75006 Paris, France; 18 Department of Internal Medicine, Hôpital Cochin, 27, Rue du Faubourg Saint-Jacques, 75679 Paris Cedex 14, France

**Keywords:** COVID-19, Primary hemostasis, Platelets, Thrombo-inflammation

## Abstract

**Background:**

Microvascular, arterial and venous thrombotic events have been largely described during severe coronavirus disease 19 (COVID-19). However, mechanisms underlying hemostasis dysregulation remain unclear.

**Methods:**

We explored two independent cross-sectional cohorts to identify soluble markers and gene-expression signatures that discriminated COVID-19 severity and outcomes.

**Results:**

We found that elevated soluble (s)P-selectin at admission was associated with disease severity. Elevated sP-selectin was predictive of intubation and death (ROC AUC = 0.67, *p* = 0.028 and AUC = 0.74, *p* = 0.0047, respectively). An optimal cutoff value was predictive of intubation with 66% negative predictive value (NPV) and 61% positive predictive value (PPV), and of death with 90% NPV and 55% PPV. An unbiased gene set enrichment analysis revealed that critically ill patients had increased expression of genes related to platelet activation. Hierarchical clustering identified *ITG2AB*, *GP1BB*, *PPBP* and *SELPLG* to be upregulated in a grade-dependent manner. ROC curve analysis for the prediction of intubation was significant for *SELPLG* and *PPBP* (AUC = 0.8, *p* = 0.046 for both). An optimal cutoff value for *PBPP* was predictive of intubation with 100% NPV and 45% PPV, and for *SELPLG* with 100% NPV and 50% PPV.

**Conclusion:**

We provide evidence that platelets contribute to COVID-19 severity. Plasma sP-selectin level was associated with severity and in-hospital mortality. Transcriptional analysis identified *PPBP/CXCL7* and *SELPLG* as biomarkers for intubation. These findings provide additional evidence for platelet activation in driving critical COVID-19. Specific studies evaluating the performance of these biomarkers are required.

**Supplementary Information:**

The online version contains supplementary material available at 10.1186/s13613-021-00899-1.

## Background

Severe acute respiratory syndrome coronavirus 2 (SARS-CoV-2) is the causative agent of the coronavirus disease 2019 (COVID-19) pandemic [1]. Severe disease is characterized by an acute respiratory distress syndrome (ARDS), respiratory failure and death in about 1% of cases [2, 3]. Most important risk factors for severe disease include age, overweight, diabetes, hypertension and history of cardiovascular disease [4, 5]. Severe and critical patients were shown to develop arterial and venous thrombotic complications, such as pulmonary embolism, stroke and myocardial infarction [6, 7]. Markers of coagulation activation, in particular increased D-dimer, fibrinogen and von Willebrand factor (vWF) levels were found to be associated with critical illness, whereas only minor changes were noted in prothrombin time and platelet counts [8–11]. In addition, autopsy series described multiple thrombosis in deceased COVID-19 patients [12, 13]. These findings suggest vascular micro-thrombotic disease as a primary factor for mortality in critically ill COVID-19 [6, 14]. Therefore, some authors supported the systematic use of curative anticoagulation upon admission to intensive care unit (ICU) [15], a strategy that has been reported to decrease mortality in severe COVID-19 [8, 16–18].

Mechanisms underlying increased thrombotic events are still unclear but accumulating evidence point to a key role for endothelial and platelet activation [19, 20]. As viral inclusions were described in endothelial cells, it has been hypothesized that endothelial cell injury and activation could drive platelet activation and subsequent coagulopathy [13, 21]. Therefore, dissecting the contribution of platelets to COVID-19 critical illness is key to understand SARS-CoV-2 infection pathogenesis and identify novel therapeutic strategies.

Platelet P-selectin is a key thromboinflammatory molecule involved in platelet activation and function. It has been demonstrated to play a crucial role in primary hemostasis by regulating platelet–leukocyte interactions, fibrin and tissue factor recruitment into platelet aggregates and thrombus formation [22]. Its soluble form, sP-selectin, is released upon platelet and/or endothelial cell activation and measurement of sP-selectin has been proposed as a reliable marker of in vivo platelet activation [23]. Moreover, sP-selectin levels have been shown to correlate with acute lung injury severity score and related death [24].

The aim of our study was to assess, in hospitalized COVID-19 patients, the ability of sP-selectin to predict requirement for mechanical ventilation and in-hospital mortality. Next, using whole-blood transcriptional data, we uncovered a platelet activation transcriptional signature associated with critical illness.

## Methods

### Cohorts

Two independent cohorts were analyzed for this study. Data used for the analysis of soluble P-selectin’s ability to predict admission to ICU were extracted from a non-interventional study that was conducted at European Georges Pompidou Hospital (Paris, France) and partially described in [25] (Cohort 1). Briefly, Cohort 1 included consecutive patients with SARS-CoV-2 infection. Inclusion criteria were patients over 18 years of age, with a proven SARS-CoV-2 infection, which presented to the emergency department with hospitalization criteria. Patients were then hospitalized into conventional wards or directly to the ICU. For all patients, baseline characteristics (demographic, treatment, clinical, cardiovascular risk factors and body mass index) and biological data were retrieved from the medical records using a standardized data collection. As a control non-COVID-19 septic ICU cohort, we included plasma samples from a previously described pre-pandemic cohort [25, 26].

Cohort 2 [27] was conducted between March 19, 2020 and April 3, 2020 in Cochin Hospital (Paris, France), in the setting of the local RADIPEM biological samples collection derived from samples collected in routine care. Inclusion criteria for COVID-19 inpatients were: age between 18 and 80 years, diagnosis of COVID-19 according to WHO interim guidance and positive SARS-CoV-2 RT-PCR testing on a respiratory sample (nasopharyngeal swab or invasive respiratory sample). Detailed clinical and immunological characterization of the cohort was previously described in [27]. Epidemiological, demographic, clinical, laboratory, treatment, and outcome data were extracted from electronic medical records using a standardized data collection form.

The severity of COVID-19 was classified at the time of inclusion based on the adaptation of the Sixth Revised Trial Version of the Novel Coronavirus Pneumonia Diagnosis and Treatment Guidance and described in [27].

### Soluble P-selectin measurement

sP-selectin quantification was performed on Cohort 1. Plasmas were collected on 0.129 M trisodium citrate tubes (9NC BD Vacutainer, Plymouth, UK). Plasma poor platelet was obtained after centrifugation twice at 2500 *g* for 15 min. PPP was frozen after a second centrifugation at 2500 *g* for 15 min and stored at − 80 °C until analysis of vascular markers. Soluble P-selectin were quantified in PPP with a Human Magnetic Luminex Assay from R&D systems (Lille, France). Data were assessed with the Bio-Plex 200 using the Bio-Plex Manager 5.0 software (Bio-Rad, Marnes-la-Coquette, France). Normalized concentration (NC) used to calculate receiver operating characteristic (ROC) area under curve (AUC) *p* values represents sP-selectin concentration in pg/mL normalized to platelet numbers (10^6^/mL).

### Gene expression analysis

Detailed methods was previously reported in Hadjadj et al. [27]. Briefly, we analyzed 100 ng (5 μL) of total RNA from each sample using the Nanostring Human Immunology kit v2 according to manufacturer’s instructions. Raw RNA counts were adjusted using five housekeeping genes selected from the 15 candidate control genes provided by Nanostring, following the geNorm method. For gene set enrichment analysis (GSEA), genes were ordered by *t*-statistic from unpaired *t* test comparing normalized RNA levels of severe vs critical patients, and then fed to the gene set enrichment algorithm (GSEA version 4.0.3, Broad Institute), along with a pathway data set built from the Nanostring Immunology panel version 2 annotation file. Parameters were set as follows: method, pre ranked gene list; number of permutations, 2000; enrichment statistic, classic; min set size, 5; max set size, 200; and all other parameters as default. Hierarchical clustering of genes belonging to the hemostasis gene set was performed using hclust with default distance matrix. Heatmap displaying genes that are upregulated in a grade-dependent manner was obtained using pheatmap (package pheatmap), with data centered to 0 and scaled to unit variance for each gene. Normalized RNA count (NRC) used to calculate ROC AUC *p* values represents adjusted RNA count (to housekeeping genes) normalized to platelet numbers (10^9^/L).

### Statistical analyses

Quantitative variables were compared among groups using Kurskal–Wallis test followed by Dunn’s post-test, while quantitative variables were compared using the χ^2^ test of independence. Correlations coefficients and *p* values were assessed using Spearman’s method. ROC AUC *p* values were determined using Hanley’s method. Optimal thresholds were determined by maximization of Youden’s index. Multivariate logistic regression was performed using death as the dependent variable, and log_10_(normalized P-selectin), age and sex as independent variables. All analyses were two-sided and a *p* value smaller than 0.05 was considered statistically significant. Statistical analyses were performed using R v. 3.4.3 (CRAN).

## Results

### Patient characteristics

Cohort 1 consisted of 60 COVID-19 patients that were admitted to European Georges Pompidou Hospital and included upon admission. Clinical and biological characteristics are previously reported in [28] and described in Table 1. Briefly, median age was 58.5 years (IQR, 49 to 72) and 76.7% were male. Patients were analyzed after a median duration of 6 days (IQR, 4 to 7) after onset of first symptoms. Interval from first symptoms on hospital admission and from first symptoms on blood sampling coincided in most patients. Fever was present in 97% of the patients, and other most common symptoms were cough (83.3%), dyspnea (71.7%), fatigue (70.0%), myalgia (40.0%) and diarrhea (26.7%). Degree of COVID-19 severity was categorized as mild-to-moderate in 35 patients (clinical symptoms associated with dyspnea and requiring a maximum of 3 L/min), severe in 10 patients (respiratory distress requiring more than 3 L/min of oxygen and no other organ failure) and critical in 15 patients (respiratory failure requiring mechanical ventilation). Eleven out of 35 patients with mild-to-moderate disease and 3/10 patients with severe disease experienced clinical worsening and required mechanical ventilation. Thrombotic events were noted in 8 patients (3 mild-to-moderate, 2 severe and 3 critical). We also included and analyzed plasma samples from a previously described pre-pandemic cohort [25, 26] as an ICU control non-COVID-19 septic ICU cohort.

**Table 1:** Clinical characteristics and laboratory findings of patients from Cohort 1

Characteristics	All patients	Disease severity			*p* value
	*N* = 60	Mild-to-moderate	Severe	Critical	
		*N* = 35	*N* = 10	*N* = 15	
Age, median (IQR), year	58.50 [49.00, 72.25]	59.00 [47.50, 74.00]	58.00 [47.25, 70.00]	58.00 [54.00, 68.50]	0.98
Male, no. (%)	46 (76.7)	22 (62.9)	10 (100.0)	14 (93.3)	**0.011**
Median interval from first symptoms	6.0 [4.0, 8.0]	6.0 [3.0, 7.0]	4.0 [4.0, 7.5]	7.0 [6.5, 9.0]	0.063
Coexisting disorder, no. (%)					
Any		18 (51.4)	5 (50.0)	11 (73.3)	0.18
COPD	1 (1.7)	0 (0.0)	0 (0.0)	1 (6.7)	0.21
Diabetes	16 (26.7)	8 (22.9)	3 (30.0)	5 (33.3)	0.72
Hypertension	27 (45.0)	14 (40.0)	5 (50.0)	8 (53.3)	0.65
Cardiovascular disease	12 (20.0)	5 (14.2)	3 (30.0)	4 (26.7)	0.26
Cancer or hemopathy	3 (5.0)	3 (8.6)	0 (0.0)	0 (0.0)	0.32
Chronic renal disease	8 (13.3)	4 (11.4)	3 (30.0)	1 (6.7)	0.21
Overweight	39 (65.0)	19 (54.3)	7 (70.0)	13 (86.7)	**0.026**
Fever on admission					
Median temperature (IQR), °C	38.20 [37.70, 38.80]	38.20 [37.80, 38.70]	38.00 [37.60, 38.35]	38.70 [37.40, 39.00]	0.53
Symptoms on admission no. (%)					
Fever	58 (96.7 )	34 (97.1 )	9 (90.0)	15 (100)	0.38
Cough	50 (83.3)	32 (91.4)	7 (70.0)	11 (73.3)	0.14
Dyspnea	43 (71.7)	21 (60.0)	8 (80.0)	15 (100)	**0.046**
Fatigue	42 (70.0)	21 (60.0)	8 (80.0)	15 (100)	0.36
Myalgia	24 (40.0)	15 (42.9)	4 (40.0)	5 (33.3)	0.87
Diarrhea	16 (26.7)	10 (28.6)	3 (30.0)	3 (20.0)	0.79
Median oxygen requirement (IQR, L/min)	3.00 [2.00, 5.00]	2.00 [1.25, 2.00]	4.00 [4.00, 5.00]	MV	–
Clinical outcomes, no. (%)					
Thrombotic event (%)	8 (13.3)	3 (8.6)	2 (20.0)	3 (20.0)	0.44
Clinical worsening requiring MV (%)	–	11 (31.4)	3 (30.0)	–	–
Death (%)	17 (28.3)	4 (11.4)	2 (20.0)	11 (73.3)	**< 0.001**
Laboratory findings on admission					
Leukocytes (IQR), × 10^9^/L	5.75 [4.47, 7.77]	5.40 [4.15, 6.85]	6.35 [5.05, 10.92]	7.60 [4.90, 11.90]	**0.041**
Neutrophils (IQR), × 10^9^/L	4.36 [3.13, 6.83]	3.74 [2.84, 5.18]	5.43 [3.89, 10.86]	6.99 [3.88, 10.48]	**0.015**
Lymphocytes (IQR), × 10^9^/L	0.85 [0.62, 1.15]	0.88 [0.71, 1.10]	0.85 [0.57, 1.25]	0.60 [0.46, 1.05]	0.19
Monocytes (IQR), × 10^9^/L	0.35 [0.24, 0.49]	0.35 [0.25, 0.48]	0.43 [0.35, 0.59]	0.26 [0.16, 0.53]	0.26
Platelets (IQR), × 10^9^/L	164 [124, 221]	165 [124, 216]	167 [135, 242]	162 [127.00, 258]	0.79
CRP (IQR), mg/L	104 [62, 168]	86 [38, 120]	117 [109, 184]	161 [98, 207]	**0.005**
Lactate dehydrogenase (IQR), U/L	–	–	–	–	
Fibrinogen (mean (SD)), g/L	5.83 [4.90, 6.50]	5.30 [4.62, 6.30]	6.80 [5.85, 7.38]	6.30 [5.65, 7.00]	**0.006**
DDimer (mean (SD)), ng/ml	999 [662, 1724]	789 [537, 1270]	1145 [759, 2142]	1170 [988, 2151]	0.05
Soluble Pselectine, pg/mL	23663 [18693, 30394]	20556 [17484, 29677]	25239 [21637, 28565]	27041 [24171, 38934]	**0.048**

Significant values (*p* < 0.05) are emphasized in bold

Quantitative variables are described as median (interquartile range) and compared using the Kruskal–Wallis test; qualitative variables are described as *n* (%) and compared using the *χ*^2^ test of independence

*COPD* chronic obstructive pulmonary disease, *CRP* C-reactive protein, *IQR* interquartile range, *MV* mechanical ventilation

Cohort 2 consisted of 50 patients with various degree of COVID-19 severity admitted to Cochin hospital and 18 healthy controls, and was described in [27]. For the purpose of the present study, only patients with whole-blood transcriptional analysis were included, i.e., 32 COVID-19 patients and 13 healthy controls. Characteristics of the patients are described in Table 2. Median age was 56 years (IQR, 51 to 65) and 78% were male, while median age of healthy controls was 59 years (IQR, 41 to 60) and 77% were male. Patients were analyzed after a median duration of 10 days (IQR, 9 to 11) after onset of first symptoms. Interval from first symptoms on hospital admission and from first symptoms on blood sampling coincided in most patients. Fever was present in 100%, and other most common symptoms were dyspnea (100%), fatigue (32%), cough (97%), myalgia (97%) and diarrhea (34%). Degree of COVID-19 severity was categorized as mild-to-moderate in 11 patients (median oxygen requirement 1.5 L/min), severe in 10 patients (median oxygen requirement 5 L/min) and critical in 11 patients. No patients with mild-to-moderate disease required admission to an ICU, while 5 out of 10 patients with severe disease were eventually admitted to the ICU. Thrombotic events were noted in 3 patients (1 severe and 2 critical).

**Table 2:** Clinical characteristics and laboratory findings of patients from Cohort 2

Characteristics	Healthy controls	All patients	Disease severity			*p* value
	*N* = 13	*N* = 32	Mild-to-moderate	Severe	Critical	
			*N* = 11	*N* = 10	*N* = 11	
Age, median (IQR), year	59.2 (45.2, 60.0)	55.6 (51.2, 64.8)	55.9 (44.7–65.1)	53.5 (48.02–61)	60.2 (54.8–71.65)	0.23
Male, no. (%)	10 (77)	24 (75)	8 (72.7)	9 (90)	7 (63.6)	0.37
Median interval from first symptoms on admission (IQR), days	–	10 (9–11)	9 (9–11)	10.5 (10–12)	9 (8–11)	0.06
Coexisting disorder, no. (%)						
Any	0 (0)	14 (44)	2 (18)	3 (30)	9 (82)	**0.006**
COPD	0 (0)	1 (3)	0 (0)	0 (0)	1 (9)	0.37
Diabetes	0 (0)	5 (16)	0 (0)	1 (10)	4 (36)	0.053
Hypertension	0 (0)	10 (31)	2 (18)	2 (20)	6 (55)	0.12
Cardiovascular disease	0 (0)	3 (9)	0 (0)	0 (0)	3 (27)	**0.042**
Cancer or hemopathy	0 (0)	0 (0)	0 (0)	0 (0)	0 (0)	–
Chronic renal disease	0 (0)	1 (3)	0 (0)	0 (0)	1 (9)	0.37
Overweight	0 (0)	2 (6)	1 (9)	1 (10)	0 (0)	0.5
Fever on admission						
Median temperature (IQR), °C	–	38.9 (38.5–39.4)	38.8 (38.2–39.5)	38.6 (38.5–39.7)	39.0 (38.5–39.4)	–
Symptoms on admission (%)						
Fever	–	32 (100)	11 (100)	10 (100)	11 (100)	
Dyspnea	–	32 (100)	11 (100)	10 (100)	11 (100)	
Cough	–	31 (97)	10 (91)	10 (100)	11 (100)	0.37
Fatigue	–	31 (97)	10 (91)	10 (100)	11 (100)	0.37
Myalgia	–	20 (63)	8 (73)	8 (10)	4 (36)	**0.082**
Diarrhea	–	11 (34)	4 (36)	5 (50)	1 (9)	**0.091**
Median oxygen requirement (IQR, L/min)						
Clinical outcomes, no. (%)						
Thrombotic events	–	3 (9)	0 (0)	1 (10)	2 (18)	0.34
Clinical worsening requiring MV	–	–	0 (0)	5 (50)	–	–
Death	–	5 (15.6)	0 (0)	0 (0)	5 (45.5)	**< 0.001**
Laboratory findings on admission						
Leukocytes (IQR), × 10^9^/L	–	6.7 (4.31–8.82)	4.71 (3.78–5.68)	7.78 (6.46–8.43)	9.38 (5.48–10.49)	**0.038**
Neutrophils (IQR), x 10^9^/L	–	5.08 (3.12–7.37)	3.25 (2.07–3.44)	5.81 (4.74–6.36)	7.69 (4.32–9.13)	**0.022**
Lymphocytes (IQR), × 10^9^/L	–	0.84 (0.56–1.13)	1.00 (0.84–1.40)	0.88 (0.57–1.12)	0.65 (0.45–0.84)	**0.031**
Monocytes (IQR), × 10^9^/L	–	0.41 (0.23–0.52)	0.40 (0.26–0.52)	0.42 (0.27–0.51)	0.33 (0.12–1.05)	0.95
Platelets (IQR), × 10^9^/L	–	249 (159–298)	166 (112–251)	229 (170–282)	313 (199–352)	**0.007**
CRP (IQR), mg/L	0.7 (0.0–0.8)	118 (55–242)	30 (14–76)	169 (136–249)	159 (109–308)	**< 0.001**
Lactate dehydrogenase (IQR), U/L	169 (155–224)	424 (346–574)	262 (196–454)	411 (396–623)	504 (426–614)	0.1

Significant values (*p* < 0.05) are emphasized in bold

Quantitative variables are described as median (interquartile range) and compared using the Kruskal–Wallis test; qualitative variables are described as *n* (%) and compared using the *χ*^2^ test of independence

*COPD* chronic obstructive pulmonary disease, *CRP* C-reactive protein, *IQR* interquartile range, *MV* mechanical ventilation

### Increased plasma sP-selectin on hospital admission is associated with disease severity and in-hospital mortality

**Fig. 1:**
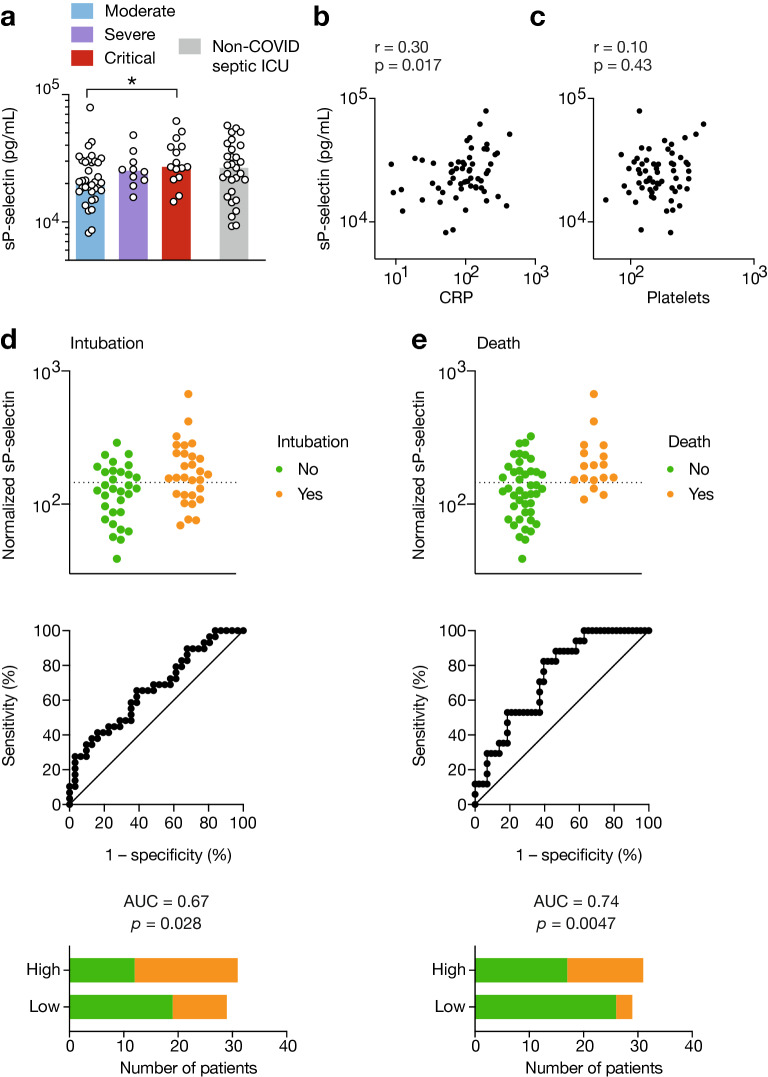
sP-selectin as a marker of COVID-19 severity and a predictor of intubation and death. **a** Soluble P-selectin was measured in plasma from patients from COVID-19 patients from Cohort 1 and plotted according to severity grade (moderate, severe or critical disease) or from non-COVID-19 septic shock patients. Groups: mild-to-moderate (*n* = 35), severe (*n* = 10), critical (*n* = 15), non-COVID-19 septic shock (*n* = 29). *p < 0.05 (Kruskal–Wallis test followed by Dunn’s post-test). **b**, **c**, sP-selectin correlation with CRP levels (**b**) or platelet counts (**c**). *r* and *p* values were determined using Spearman’s correlation test. **d**, **e**, sP-selectin normalized to platelet counts as a predictor of intubation (**d**) or death (**e**). ROC curves with area under the curve (AUC) and associated *p* values are shown. Optimal threshold was determined by maximization of Youden’s index, and represented by a dashed line. Bar graphs indicate the number of patients in each group depending on normalized sP-selectin levels with respect to the optimal threshold. Groups: no intubation (*n* = 31), intubation (*n* = 29), no death (*n* = 43), death (*n* = 17)

We measured plasma sP-selectin in Cohort 1. Increased plasma sP-selectin levels at admission was significantly associated with critical disease (Fig. 1a) in COVID-19 patients, albeit at similar levels compared to non-COVID-19 septic ICU patients (Fig. 1a and [25, 26]). Moreover, sP-selectin levels were positively correlated with inflammatory parameters, i.e., CRP levels (*r* = 0.30, *p* = 0.017, Fig. 1b), but not with platelet count (*r* = 0.1, *p* = 0.43, Fig. 1c).

We next evaluated the ability of sP-selectin, normalized to platelet counts, to predict intubation (Fig. 1d) or in-hospital mortality (Fig. 1e) using ROC curves. We found that outcomes were significantly associated with higher sP-selectin values (AUC = 0.67, *p* = 0.028 for intubation and AUC = 0.74, *p* = 0.0047 for in-hospital mortality). An optimal cutoff value of 150 normalized concentration (pg/mL normalized to 10^6^ platelets/mL) was predictive for intubation with 66% sensitivity, 61% specificity, 66% negative predictive value (NPV) and 61% positive predictive value (PPV), and death with 82% sensitivity, 60% specificity, 90% NPV and 55% PPV. Multivariate logistic regression after adjustment for age and gender confirmed the association of elevated sP-selectin with in-hospital mortality (OR = 72.0 (2.99–3600.00), *p* = 0.016). Clinical follow-up of COVID-19 patients initially non-critical (moderate and severe patients) also found that higher sP-selectin values were associated with worst clinical outcomes, albeit without reaching significance (AUC = 0.66, *p* = 0.086 for intubation and AUC = 0.70, *p* = 0.12 for in-hospital mortality) (Additional file 1: Figure S1). Interestingly, the predictive potential of normalized sP-selectin in non-COVID-19 septic ICU patients from a pre-pandemic cohort was not significantly associated with in-hospital mortality (AUC = 0.54, *p* = 0.71) (Additional file 2: Figure S2).

Together, these data suggested that sP-selectin is associated with in-hospital mortality in COVID-19 patients and may identify patients at higher risk of critical illness.

### Critically ill COVID-19 patients display a platelet activation transcriptional signature

**Fig. 2:**
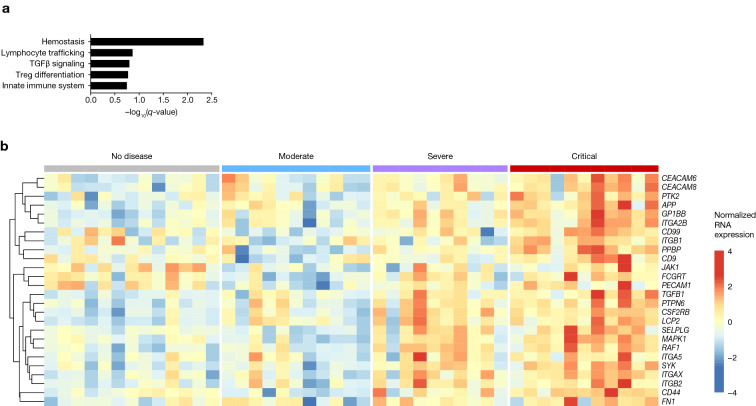
Unbiased transcriptomic analysis reveals primary hemostasis-related genes as upregulated in critically ill COVID-19 patients. RNA from patients of Cohort 2 was extracted from whole blood and RNA counts of 594 genes were determined by direct probe hybridization using the Nanostring nCounter Human Immunology v2 kit. **a** Gene-set enrichment analysis was performed after ranking genes according to their differential expression in severe vs critical patients. Shown are pathways significantly enriched (false discovery rate < 0.2). **b** Heatmap representation of genes of the primary hemostasis pathway that are upregulated in a severity grade-dependent manner (determined by hierarchical clustering). Up-regulated genes are shown in red and down-regulated genes in blue. Groups: healthy controls (*n* = 13), mild-to-moderate (*n* = 11), severe (*n* = 10) and critical (*n* = 11)

**Fig. 3:**
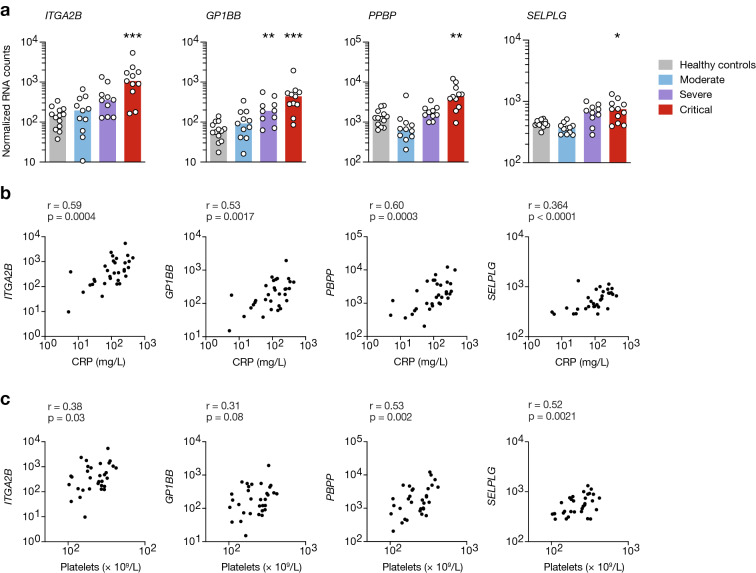
Platelet activation gene expression profile in critically ill patients. **a** Normalized RNA expression of *ITGA2B*, *GP1BB*, *PPBP*, *SELPG* depending on severity grade. *p < 0.05, **p < 0.01, ***p < 0.001 (Kruskal–Wallis test followed by Dunn’s post-test). **b**, **c** Gene expression correlation with CRP levels (**b**) or platelet counts (**c**). *r* and *p* values were determined using Spearman’s correlation test. Groups: healthy controls (*n* = 13), mild-to-moderate (*n* = 11), severe (*n* = 10) and critical (*n* = 11)

We previously performed a whole-blood immunological transcriptional characterization on 32 patients with laboratory-confirmed COVID-19 displaying various disease severity (cohort 2) [27]. Using an unbiased gene set enrichment analysis comparing different COVID-19 severity grades, we determined a gene signature that specifically discriminated severe from critical illness. GSEA pathway enrichment analysis identified this signature as related to primary hemostasis (Fig. 2a). Hierarchical clustering identified genes that were upregulated in a grade-dependent manner, thereby driving the primary hemostasis signature in critically ill patients (Fig. 2b). These genes included *ITG2AB*, *GP1BB*, *PPBP* and *SELPLG* (Fig. 3a), which recapitulated multiple steps of platelet activation [29, 30]. Importantly, and similarly to sP-selectin, RNA levels more strongly correlated with CRP level (Fig. 3b) than with platelet counts (Fig. 3c).

**Fig. 4:**
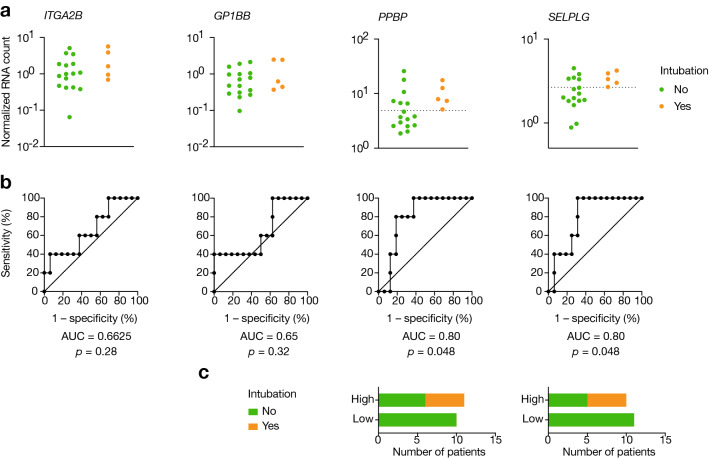
Prediction potential of hemostasis genes for mechanical ventilation. **a** RNA levels of non-severe patients at admission depending on their future aggravation, normalized to platelet counts. Dashed lines represent optimal threshold obtained by maximization of Youden’s index for significant AUCs. **b** Associated ROC curves, with area under the curve (AUC) and associated *p* values. **c** Bar graphs indicating the number of patients in each group depending on normalized RNA levels of a single gene with respect to the optimal threshold. Groups: no intubation (*n* = 16) and intubation (*n* = 5)

We next evaluated the ability of these genes to predict clinical outcome after normalization with platelets. Among patients that did not initially require critical care upon admission (i.e., moderate and severe patients, *n* = 21), 5 (24%) presented respiratory failure afterwards requiring mechanical ventilation (Table 2). *PPBP* and *SELPLG* were found to be the best predictor of clinical worsening (AUC = 0.8, *p* = 0.048 for both outcomes) (Fig. 4a–c). An optimal cutoff value for *PBPP* of 5 normalized RNA counts (NRC, normalized to 10^6^ platelets/mL) was predictive of intubation with 100% sensitivity, 63% specificity, 100% NPV and 45% PPV, while a cutoff value of 2.65 NRC for *SELPLG* was predictive of intubation with 100% sensitivity, 69% specificity, 100% NPV and 50% PPV. Overall, these markers provide excellent negative predictive values, with no patient below the threshold value requiring future mechanical ventilation.

## Discussion

Dysregulated hemostasis is emerging as a key factor in COVID-19 pathogenesis and severity. The present study provides new insights into the contribution of platelets to disease severity with the identification of a unique hemostasis signature in critically ill patients. We identified sP-selectin as a soluble marker associated with in-hospital mortality, and elevated *SELPLG* and *PPBP* RNA levels as strong negative predictors of mechanical ventilation in hospitalized patients.

Elevated sP-selectin levels promote leukocyte–endothelial and leucocyte–platelet adhesion [31], release of citrullinated histones and neutrophil extracellular traps (NETs) formation [32], promoting formation of platelet–neutrophil or –monocyte aggregates via binding of P-selectin to its ligand PSGL-1 (encoded by *SELPLG*) expressed on the surface of leukocytes [33]. Accordingly, elevated sP-selectin and increase in platelet–monocyte aggregates and have been recently described in COVID-19 patients [33–35], the latter being effectively blocked by platelet P-selectin neutralization [33]. Our study supports these findings and highlights the key role of platelet activation in critical patients.

Importantly, while signatures related to innate immune activation and impaired interferon activity distinguished mild-to-moderate disease from severe and critical disease [27], we show that only a primary hemostasis-related gene signature could further distinguish severe from critical patients (Fig. 2a), especially the specific platelet chemokine-encoding gene *PPBP/CXCL7* and *SELPLG,* encoding for the P-selectin glycoprotein ligand-1 (PSGL-1), which also predicted clinical outcome. Pro-platelet basic protein (PPBP), also known as CXCL7, is the most abundant platelet chemokine [29, 36], expressed within platelets as an inactive precursor and activated after cleavage during thrombus formation by enzymes released by neutrophils. PSGL-1 is expressed by leukocytes, allowing the formation of platelet–leukocyte conjugates and adhesion of leukocytes to activated endothelium through its interaction with P- or E-selectin [29, 37]. PBPP/CXCL7 has been shown to be essential for neutrophil migration into the thrombus [38], and the formation of platelet–neutrophil aggregates and murine models of acute lung injury showed that deletion of *PPBP/CXLC7* protected the mice from lung disease [36], as well as blocking platelet activation [39]. Overall, our data support a model, whereby platelet activation induces PPBP/CXCL7 release and P-selectin upregulation followed by neutrophil attraction into sites of injury and the formation of platelet–leucocyte aggregates, precipitating organ injury and failure.

In addition to their role in primary hemostasis, platelets are an integral part of the immune response to pathogens. The functional interdependence and the coordinated activation of both processes, designated as thrombo-inflammation, may drive adverse effects, such as thrombosis, multiple organ failure and death [40]. We support the hypothesis that deregulated primary hemostasis, in association to inflammation, could drive COVID-19 progression into critical disease. Viral infection and sepsis models have been associated with platelet gene expression and functional alterations [41, 42]. Supporting this generalizable thrombo-inflammation model, we found similar elevated sP-selectin in non-COVID septic ICU patients. However, some unique features may characterize SARS-CoV-2-mediated platelet dysregulation. Authors have shown that sP-selectin seemed to be more strongly associated COVID-19-related in-hospital mortality [43, 44]. Frazer et al. [45] found significantly elevated sP-selectin in ICU patients in comparison to non-COVID-19 ICU patients at day 3 post-admission (and not at days 1 and 2), that persisted until day 7. This finding suggests that persistent platelet activation may characterize COVID-19-associated coagulopathy. In addition, Manne et al. showed that platelet gene expression profile was unique in COVID-19 patients in comparison to H1N1 pandemic patients. Altogether, these data demonstrate that COVID-19 critical patients share important similarities with other critical sepsis, but some features may uniquely contribute to platelet activation during SARS-CoV-2 infection.

Prediction of illness trajectories after the onset of symptoms is difficult but remains critical. Clinical and biological factors have been shown to predict poor outcome, including arterial blood gazes values (e.g., hypoxemia, hypercapnia, *P*/*F* ratio, lactate), inflammatory markers (e.g., CRP, IL-6), coagulation markers (e.g., VWF, D-dimer) and cellular blood composition (e.g., neutrophil-to-lymphocyte ratio). These factors have led to the development of prognosis scores to predict critical illness [46]. Our findings suggest that markers of dysregulated hemostasis could be evaluated for their predictive prognostic value.

Finally, these findings suggest that a subset of patient may benefit from drugs preventing platelet activation, such as antiplatelet agents or P-selectin inhibitors [47], which have also been proposed in other septic settings [48, 49]. These anti-platelets agents in combination with anti-inflammatory drugs (glucocorticoids, anti-IL-6) could have substantial impact on thrombo-inflammation.

The benefit of anti-platelet agents is, however, challenged by recent findings from Althaus and coll. [50] which proposed that the induction of a procoagulant platelet subpopulation may be a key driver of critical COVID-19. Procoagulant platelets displayed increased P-selectin, integrin αIIb and GPIbα expression on their surface [51], in line with biomarkers (sP-selectin, *PPBP*, *SELPLG*) and signature (*ITG2AB,* *GP1BB,* *PPBP* and *SELPLG*) we identified. However, in this scenario, the benefits of aspirin and P2Y_12_ inhibitors would be limited; blockade of P2Y_1_ and P2Y_12_ does not inhibit procoagulant platelets activation [52] and pretreatment with aspirin has minimal effect on their generation [53]. In contrast, Manne et al. [35] did not find evidence for an increased procoagulant platelet subpopulation, and an additional study concluded that procoagulant platelets may, on the contrary, have a protective effect during SARS-CoV-2-related pneumonia [54]. Therefore, the mechanisms underlying platelets activation, and subsequently, the potential benefit of anti-platelet therapy remain controversial. The REMA-CAP trial is currently investigating the use of aspirin or a P2Y_12_ inhibitor (clopidogrel, prasugrel, or ticagrelor) [55], and results from the RECOVERY trial on aspirin will soon be unraveled [56]. So far, one retrospective trial suggested a potential benefit of aspirin use on critical care and in-hospital mortality [57].

Our study has some limitations. We used two independent cohorts with a small sample size from two centers. The two cohorts were not homogenous in regard to timing of measurements and severity of patients. In addition, the study was not designed as a longitudinal study, so no sequential measurement was available. Indeed, sP-selectin association with clinical outcome was moderate and serial measurement over time may optimize its outcome prediction performance. Moreover, longitudinal analysis of sP-selectin levels may provide insights on the causal role of platelets activation in driving critical disease. Our transcriptomic analysis was performed on whole-blood RNA, so we cannot evaluate the contribution of each cell population to hemostasis dysregulation. Separate transcriptional profiling of platelets vs other circulating populations and analysis of cell expression phenotypes (P-selectin, phosphatidyl-serine exposure) and evaluation of platelets functions may provide further insights into the contribution of each population to thrombo-inflammation and dissect the mechanisms of platelet–neutrophil or platelet–monocyte aggregates.

Overall, this exploratory study sheds light onto the role of thrombo-inflammation in critical patients. We identified platelet activation markers sP-selectin, *SELPLG* and *PPBP/CXCL7* as potential biomarkers of critical worsening. Additional studies dedicated to evaluate the predictive performance of these biomarkers are required to both validate our findings and optimize their ability to predict progression to critical disease.

## Supplementary Information

**Additional file 1: Figure S1.** sP-selectin as a marker of later requirement for mechanical ventilation or in-hospital mortality in mild-to-moderate and severe COVID-19 patients sP-selectin normalized to platelet counts as a predictor of intubation (left) or death (right). Each dot represents one patient (upper panel). ROC curves with area under the curve (AUC) and associated *p* values are shown (lower panel). Groups: no intubation (*n* = 31), intubation (*n* = 14), no death (*n* = 39), death (*n* = 6).

**Additional file 2: Figure S2**. sP-selectin is not associated with death in non-COVID-19 septic ICU patients. sP-selectin normalized to platelet counts as a predictor of intubation (left) or death (right). Each dot represents one patient (upper panel). ROC curves with area under the curve (AUC) and associated *p* values are shown (lower panel). Groups: no death (*n* = 13), death (*n* = 16).

## Data Availability

All data are available upon reasonable request.
