# Supplementary figures and images for: Platelet activation in critically ill COVID-19 patients
Source: Ann Intensive Care. 2021 Jul 17;11:113. doi: 10.1186/s13613-021-00899-1 (PMC8286043; doi:10.1186/s13613-021-00899-1)

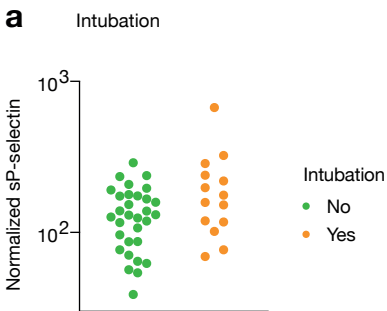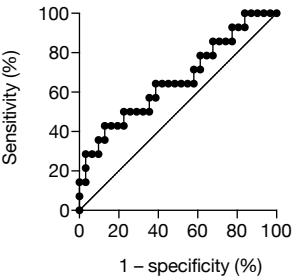

AUC = 0.66

$p = 0.086$

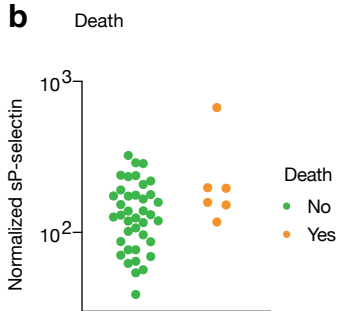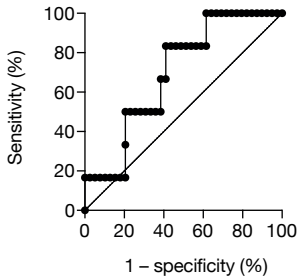

AUC = 0.70

$p = 0.12$

Supplement: Supplementary file 1 — Additional file 1: Figure S1. sP-selectin as a marker of later requirement for mechanical ventilation or in-hospital mortality in mild-to-moderate and severe COVID-19 patients sP-selectin normalized to platelet counts as a predictor of intubation (left) or death (right). Each dot represents one patient (upper panel). ROC curves with area under the curve (AUC) and associated p values are shown (lower panel). Groups: no intubation (n = 31), intubation (n = 14), no death (n = 39), death (n = 6). [file 13613_2021_899_MOESM1_ESM.pdf]

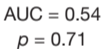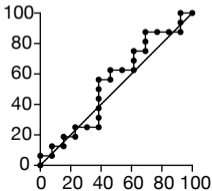

Supplement: Supplementary file 2 — Additional file 2: Figure S2. sP-selectin is not associated with death in non-COVID-19 septic ICU patients. sP-selectin normalized to platelet counts as a predictor of intubation (left) or death (right). Each dot represents one patient (upper panel). ROC curves with area under the curve (AUC) and associated p values are shown (lower panel). Groups: no death (n = 13), death (n = 16). [file 13613_2021_899_MOESM2_ESM.pdf]
